# The Framing Preference for Large and Increasing Components in Static and Dynamic Descriptions

**DOI:** 10.3389/fpsyg.2021.720427

**Published:** 2021-11-18

**Authors:** Henk Pander Maat, Ben Staal, Bregje Holleman

**Affiliations:** ^1^Utrecht Institute for Linguistics OTS, Utrecht University, Utrecht, Netherlands; ^2^Department of Language, Literature and Communication, Utrecht University, Utrecht, Netherlands

**Keywords:** attribute framing, reference point, task effect, profiling, markedness

## Abstract

Describing sets in terms of a two-valued variable, either value can be chosen: exam results may be referred to by pass rates or fail rates. What determines such framing choices? Building on work by McKenzie and colleagues on reference points in the production and interpretation of framed information, we investigate two determinants of frame choice. One is that speakers tend to focus on the component that has increased vis-à-vis a previous state, the other is the tendency to choose the component larger than 50%. We propose to view reference points as pointing to different kinds of communicative relevance. Hence the use of the previous state and the 50% reference points by speakers is not just a function of the information, but is co-determined by a communicative cue in the context: the question being asked about this information. This line of thought is supported by two experiments containing items offering two-sided distribution information at two points in time. Our first experiment employs a static task, requiring a description of the most recent situation. The second experiment uses a dynamic task, asking participants to describe the development between the two time points. We hypothesize that in static tasks the component size is the strongest frame choice determinant, while in dynamic tasks frame choice is mainly driven by whether a component has increased. The experiments consist of 16 different scenarios, both with symmetrical contrasts (i.e., *dogs* vs. *cats*) and with asymmetrical ones (i.e., *winning* vs. *losing*). Both experiments support the hypotheses. In the static task, the size effect is the only consistent effect; in the dynamic task, the effect of direction of change is much larger than that of size. This pattern of differences between size and change effects applies across symmetrical and asymmetrical contrasts. Our experiments shed light on cognitive and communicative regularities involved in the production of framed messages: people do tend to prefer larger and increasing components when choosing a frame, but the relative strength of both these preferences depends on the communicative task.

## Introduction

### Two Motivations Steering Framing Choices

Suppose elections have just been held and votes are being counted. The Liberal Party and the Social Democrats are heading to be equally large (30 seats in parliament), larger than any other party. How would journalists frame the results? Probably in terms of the two large parties, and not in terms of the other, smaller ones. Being larger, the Liberals and the Social Democrats are essential in terms of the upcoming government formation. The implicit question answered here is “what does the new political landscape look like?” But let us now take into account that the Liberals have grown substantially (9 seats in parliament) whereas the Social Democrats have lost 3 seats. Hence there is another, and equally relevant question: “how has the political landscape changed?” Journalists focusing on this question will probably highlight the progress made by the Liberals.

This article is not about political communication, but about framing. In many language-use situations, a state of affairs needs to be described which is two-sided. For instance, there is a proportion of men and a complementary proportion of women, or there is a chance of success as well as a complementary chance of failure. Often, such distributional information is presented for various points in time. How do language-users decide which frame to use in reporting them? Two options have been introduced in our election example: we might focus on the current situation, or alternatively, on what has changed. These two implicit questions may lead to different framing preferences, favoring either the largest component (in the current situation focus) or the increasing component (in the change focus). We thus aim to show how the understanding and representation of framed information is fine-tuned to the communicative task at hand. Furthermore, framing preferences may differ for symmetrical and asymmetrical word pairs. In asymmetrical contrasts (such as *winning* vs. *losing*), the conventional description of a situation uses the unmarked pair member (here *winning*), as the unmarked goal orientation is to *win*. There is no such orientational differentiation in symmetrical contrasts. We will assume that framing choices in asymmetrical pairs are more asymmetrical than in symmetrical pairs. However, we expect both kinds of pairs to be sensitive to the differences between static and dynamic communicative tasks.

### Reference Points Are Involved in Attribute Framing Choice and Interpretation

Attribute framing phenomena are a particular case of the vast number of construal choices available to language users (Langacker, 2013). In this paper we ask what determines the choice between framing alternatives, building on work by McKenzie and others on *reference points*. To introduce this notion, we need some background on attribute framing research. Many attribute framing studies concentrate on the difference between positive or negative framing options. For instance, one could say that the *success rate* of a course is 60%, or that the *failure rate* of the same course is 40%. While these descriptions seem to be true in exactly the same circumstances, they evoke different responses: speaking of the success rate generally yields more positive evaluations of the course than speaking of the failure rate, and leads to a stronger inclination to attend the course. These attitudinal and behavioral effects of attribute framing are known as the “valence-consistent shift” (Levin et al., 1998; Schneider et al., 2005). On the assumption that the two expressions are equivalent, the valence-consistent shift is considered to be an irrational behavior of language users.

But to what extent are the expressions really equivalent? McKenzie and Nelson (2003) and Sher and McKenzie (2006) distinguish between logical and informational equivalence, arguing that the two frames may be equivalent logically, but not informationally. More specifically, they say that speakers are more likely to describe a proportion in terms of component X (e.g., success rates) when X exceeds a reference point proportion than when X is below this reference point. This reference point is interesting in that it can be linked to insights from linguistic pragmatics. First, it may offer a way to characterize the relevance, i.e., the “news value” of framed utterances: that is, the fact that a value exceeds the expectation or some earlier state is exactly the point of the utterance. Second, reference points allow speakers to choose some frame, and hence to make their utterance as concise as possible. After all, given the complementarity of the components, a two-sided utterance would be needlessly long; profiling one and backgrounding the other component allows for efficient communication. Hence framing enables speakers to deliver optimally relevant utterances (Wilson and Sperber, 2008), keeping in mind that relevance is a function of both cognitive effects (here: updating your reference point) and processing effort (here: not having to process more information than necessary).

Another linguistic pragmatic notion relevant for reference points is that speakers and hearers closely cooperate in accomplishing pragmatic effects (Grice, 1975; Levinson, 2000; Wilson and Sperber, 2008). In other words, when frame choice is actually organized around reference points, these will probably be drawn on both in text production and in text interpretation. This two-sidedness finds support in the work of McKenzie and Nelson (2003), who conducted both a speaker study (a frame production task) and a listener study (a frame interpretation task). In the production study, speakers were asked to select a frame (e.g., *half full* vs. *half empty*) to describe a glass of water that had been either empty or full to start with; in the interpretation study, readers read a framed description and were asked to make an inference about the previous state of the glass “given the way the object is described now.” Sher and McKenzie (2006) used the same scenario for a behavioral experiment: speakers were presented with two cups, one full and one empty. They were asked to pour some water from the first into the second cup and then to hand over a *half full* cup (first condition) or a *half empty* cup (second condition). Both experiments supported the hypothesis that speakers tend to profile the proportion that has increased, while hearers tend to assume that the profiled proportion has increased. For instance, in the pouring experiment, the *half full* cups handed over were more often the cups recently filled than the *half empty* cups were.

This orientation on increases beyond a reference point may be deeply rooted in our cognitive systems, as McKenzie and Nelson (2003): 597 note.

“(…; English) speakers appear to have a general tendency to use terms that correspond to the label (or pole) that has increased. For example, a person whose height has increased is usually referred to as *taller*, not *less short*, whereas a person whose height has decreased (i.e., shortness has increased) is usually referred to as *shorter*, not *less tall*. Note further that there is no morpheme in English that is analogous to the suffix *-er* to indicate that a dimension has decreased, which also seems to imply that increasing labels or poles have a special status.”

The previous state reference point effect is not restricted to the English antonym pair *full/empty*. Holleman and Pander Maat (2021) report on a successful replication of the Sher and McKenzie (2006) “pouring experiment” in Dutch. Furthermore, they present a conceptual replication of the effect in a paper-and-pencil test with 16 items, using a variety of descriptors and scenarios. All scenarios contained an attribute-framed statement about the distribution of a two-valued variable in a certain year, such as the proportions of *healthy* and *unhealthy* fats in Dutch supermarket products. Half of the participants saw the statement phrased in terms of the first frame component, the other half received the complementary statement. Participants were then asked about the distribution some previous year: did they think the frame component had been lower this earlier year (i.e., it has increased) or higher (i.e., it has decreased). 58% of the choices went to increasing proportions, regardless which component had been framed, and regardless the percentage used in the scenario (the percentage range was 12–88).

Other support for the reference point effect in framing tasks comes from experiments by Honda et al. (2018), carried out in Japanese. They first replicated the McKenzie and Nelson (2003) experiment in which the reference point was overtly presented, i.e., by means of a story about a glass that was previously fuller or emptier. They added experiments in which the reference point was not verbalized but only primed. This was done in an apparently unrelated first task in which the participants estimated the contents of a nearly empty and a nearly full glass on the basis of photos; in a later experiment, the first task was only to evaluate the photos. The second task then asked for a framed description of a half-full glass. Participants primed with pictures of nearly empty glasses more often called the half-full glass *full* than those having seen a picture of a nearly full glass. When asked to provide a reason for their choice, participants were largely unaware of the role of the prime. Honda et al. (2018) conclude that it is the activation of a (low or high) reference point by itself that counts, not the way it is generated (by an explicitly chronological scenario, or by “accidental” priming).

Interestingly, this orientation on increases vis-à-vis some earlier state might point to a general human cognitive functionality. Monitoring changes in the environment is a crucial activity for any living organism, as successfully anticipating on opportunities and threats drastically increases its chances of survival and well-being (Poli, 2010, 2019; Seligman et al., 2013). Hence for both information processing and communication, it makes sense to focus on the relation between the previous and the current situation. After all, monitoring as an activity is intrinsically more focused on changes in parameter values than on absolute values. This perspective readily explains why phenomena increasing in size or frequency will receive special attention: they carry a promise of future importance.

Besides the direction of change for a component, McKenzie and associates suggest a second driver of framing choices: the size of the component to be described. In Sher and McKenzie (2006) Experiment 4, participants flip coins. When asked to describe the results, participants prefer to frame them in terms of the largest component (with no preference for a head or tails frame). The authors note that “a tendency to describe things in terms of what has increased relative to the reference point should, when the reference point is parity, favor majority descriptions” (Sher and McKenzie, 2006: 14).

Another relevant study is McKenzie et al. (2001). They invited participants to produce conditional explanations for a set of dichotomous event-outcome pairs. For instance, the participants are presented with the data for five applications to a prestigious college, in which the one applicant with a high SAT score is accepted, while the others with average scores are rejected. When asked to frame a conditional statement explaining these data, participants tend to frame them in terms of the rare combination (high score – acceptance) instead of the common combination (average score – rejection). This preference to choose the rare combination is also maintained in scenarios in which acceptance is common and rejection is rare. This tendency persists when the participants were asked not to explain, but to describe the outcome-event relation. Generally then, causal descriptions and explanations focus on rare events, not frequent ones. However, in the last experiment participants were also asked to summarize each variable *by itself*, using a single phrase with either the word *some* or *most*. It turned out they overwhelmingly used *most* in their summaries. So in summarizing isolated events, the focus is on what happens in the majority of cases.

The majority preference is based on a parity reference point, which may be the result of scenario-specific expectations, as in the coin tossing situation, and many gender distribution situations. But it also recommends itself as the default option in the absence of scenario knowledge. A majority description profiles the more representative value of some variable; hence it covers more set members than the corresponding minority description, in the same number of words. In the absence of more specific considerations, the majority description seems the optimally relevant one for a distribution at a single point in time. So just like exceeding the previous state reference point, exceeding the parity reference point corresponds to a particular kind of relevance for framed utterances.

In short, we follow McKenzie and colleagues in relating attribute framing choices in non-causal descriptions to reference points. Two kinds of reference points have been introduced: the previous-situation reference point leads to a preference to profile increasing proportions, while the parity reference point favors a frame in terms of the majority proportion. Often, both preferences are potentially at stake, as we often have information about the distributions at various points in time. *How are frame producers to weigh the two preferences in such situations? Will small increasing proportions be framed, or large decreasing ones instead?*

We assume that framed descriptions are first and foremost communicative utterances that need to be relevant in some context. Hence we hypothesize that the strength of the two preferences depends on the question addressed by the description: does this question focus on the current situation or on the development leading to that situation? Section “Different Description Tasks May Invoke Different Reference Points” will elaborate on this task variation.

We will extend earlier work in two other ways as well. Many framing studies focus on fairly specific contrasts. Lots of work has been done on the *full-empty* contrast regarding glasses; this contrast is quite unique, both in its concreteness and in its relation with the action verbs *filling* and *emptying*. Possibly, this relation leads to especially strong previous state inferences. In this study, we will use 16 different scenarios, most of which are not related to action verbs and are likely to be less vivid. The second extension is that many studies use asymmetrical contrasts only, while we will examine symmetry effects by adding it as an experimental variable. This is elaborated on in section “Asymmetrical Contrasts Add an Extra Preference.”

### Asymmetrical Contrasts Add an Extra Preference

Attribute framing contrasts derive from antonym pairs (i.e., semantic opposites). Many antonym pairs are asymmetric; linguists call one pair member unmarked and the other marked. Such markedness differences apply to adjective pairs such as *good-bad, tall-short*, and *full-empty* (see Lehrer, 1985, 2002), but also to verb pairs like *winning-losing* and noun pairs such as *success-failure*. Asymmetry in contrasts can arise due to several mechanisms:

1. Goal orientation: in contrastive pairs, the word relating to the goal of a certain action or situation is usually the unmarked pair member, whereas the opposite is marked. E.g., it is more marked to describe performance by giving the number of lost matches than by giving the number of matches won.
2. Evaluative direction: positive pair members are usually unmarked, whereas their negative counterpart is marked, e.g., *good* vs. *bad*. This affects interpretations. To say something is *good*, can imply a positive evaluation as well as a more or less neutral one, whereas the marked choice *bad* is a negative qualification.
3. Perceptive saliency: in contrastive pairs, the word relating to a visible amount of something is usually unmarked, whereas the contrast is marked, e.g., *tall* is unmarked, *short* is marked, and it’s more “normal” to describe something in terms of its length (“he’s six feet tall”) than it is in terms of its shortness (“he’s six feet short”).
4. A specific case of markedness is gender markedness: usually the female reference to a species or a profession is more marked than the male reference. E.g.: a *painter* can be male or female, whereas *paintress* refers to women painters only; *dog* refers to a general species, and *bitch* to the female subtype only.

Almost always, the unmarked pair member will be used in a wider range of contexts. That is, performances are more often assessed in terms of the number of *successes* than in the number of *failures*; and talking about glasses we more often say how *full* than how *empty* they are.

Most framing production studies ignore the symmetry factor, although its importance can be gleaned from the presented data. For instance, McKenzie and Nelson (2003, experiment 1) report how often their participants choose *full* vs. *empty* when describing glasses in different scenarios (i.e., half of the glasses had been filled, others had been emptied). When summing the frequencies over both scenario’s for glasses ending up half-full, 59.5% of the participants chose the *full*-frame; for one-quarter-full glasses, 76.5% did; and for three-quarter-full 81.5% chose the *full*-frame. This reveals a preference for the unmarked frame *full*, apart from the preference to describe the glass in terms of the frame that has increased compared to a previous state, and apart from how full the glass actually is.

Markedness does not only affect frame production, but frame interpretation too. Holleman and Pander Maat (2009) have demonstrated that marked pair members convey stronger evaluations than unmarked members. And in the pouring experiment of Holleman and Pander Maat (2021), more participants asked to hand over the empty glass chose the emptied one (84%), than the participants asked for the full glass chose the filled one (60%). Likely, the more outspoken interpretations elicited by marked pair members may help explain the production preference for unmarked members.

For our present purposes, this means we hypothesize that our asymmetrical items will probably show a larger asymmetry in choices, as one of the members benefits from the unmarkedness preference, which preference is lacking in the symmetrical items. In other words, the unmarkedness preference comes on top of the reference point related preferences effects discussed above. We do not expect reference point effects to be eliminated in asymmetrical context, but we will explore whether they are softened.

### Different Description Tasks May Invoke Different Reference Points

**FIGURE 1:**
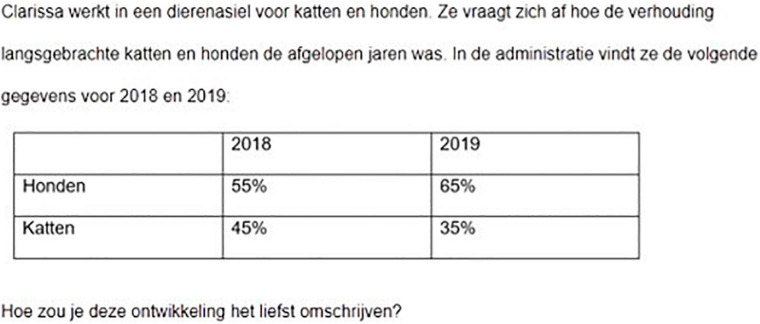
Screenshot of a symmetrical scenario used in our study (taken from experiment 2). Translation: Clarissa works in an animal shelter for cats and dogs. She wonders how many cats and how many dogs were brought into the shelter in the past years. In the computer, she finds the following data for 2018 and 2019 […]. How would you prefer to describe this development?

So far we have seen several ways in which reference points may be introduced in the frame production situation: using a scenario overtly containing the reference point, or by (implicitly) priming the reference point. In contrast, this study focuses on the reference point implications of the frame production task, i.e., the communicative goal of the framed utterance. Although frame production tasks may also focus on persuasion [e.g., see Van Buiten and Keren (2009) who asked their participants to recommend either a risky or a riskless option], we confine ourselves to description tasks and vary the descriptive focus, which may be either dynamic or static. Compare the scenario in Figure 1.

Our study consists of two experiments. In both experiments we use the same scenarios, but between experiments we vary the communicative task. In experiment 1, we presented our participants with a static description task, asking *How would you prefer to describe the situation in 2019?* Following common usage, the two points in time were presented horizontally in a table. The participants were then offered a statement about 2019 in which they had to choose between the percentage of cats and the percentage of dogs. That is, the static task requires a choice between the cells in the last column of the scenario table; participants may describe the 2019 situation by saying *the percentage of dogs is 65%*, or *the percentage of cats is 35%*. The static task is the standard task used in the existing work our study is building on to.

However, in Experiment 2 we used an alternative, *dynamic* description task: *How would you prefer to describe the development presented here?* In their answer, the participants could either refer to the decreasing percentage of dogs or to the increasing percentage of cats. That is, the dynamic task requires the participant to select one of the rows in the scenario table to report on, by either saying *the percentage of dogs goes to 65%*, or by saying *the percentage of cats goes to 35%*.

The difference between dynamically-oriented and state-oriented descriptions may be explained in terms of a visual analog. Since state-oriented descriptions focus on the containment relation between a subset and the entire set in a single distribution, the nearest visual analog of state descriptions seems to be a pie chart. In contrast, dynamic descriptions focus on the trend leading from one distribution to the other. Its visual analog is a single line graph. Shah et al. (1999) and Tversky et al. (2000) have shown that lines are more easily seen to convey trends than bars. Meyer et al. (1997) conclude the same when comparing line graphs with tables. In the context of our experiments, the core informational content itself is kept orientationally neutral, in that tables lend themselves to various processing perspectives (Meyer et al., 1997; Kauffman and Kiewra, 2010).

In our setup, the task instruction is verbally conveyed, followed by a corresponding response template. As shown above, our questions and response templates are quite explicit on the underlying questions [see Van Kuppevelt (1995) on implicit and explicit questions indicating utterance topics]. There is a large literature documenting the profound context-sensitivity of language comprehension in general (Hauser, 2017) and question answering in particular (Conrad et al., 2014): language users try to heed the concerns of their interlocutors, which are inferred from subtle wording features of their utterances. Given the robustness of these context effects, we assume that the static or dynamic nature of our question-template combinations will cue the participant to prioritize a certain reference point. Our study follows the “situated social cognition” approach (Smith and Semin, 2004) in assuming that cognitive processes are fine-tuned to support adaptive action in some context, including communicative actions. For our experiments this means that the understanding and representation of framed information using reference points will be fine-tuned to the communicative task at hand.

More generally, our study contributes to the understanding of two determinants of frame choice: the distributions talked about; and the question being asked about these distributions in a particular communicative context. We hypothesize that frame choice can partly be explained by intrinsic properties of the distribution presented in the items: (1) largest components will be framed more often than smaller components; and (2) increasing components will be framed more often than decreasing components. But framing choices cannot be explained solely by the intrinsic properties of the distribution. There is also an effect of asymmetry, in (3) that asymmetrical contrasts will show more choices for the unmarked component compared to the marked one. Furthermore, the communicative task steers the selection of the component to be profiled in the utterance. When the profiling choices would primarily respond to representational properties of the information at hand, i.e., to the size and direction of change salience of certain components, the strength of the two preferences will remain unaffected by the nature of the frame production task. However, we expect the adopted communicative perspective to determine the relative strengths of the two reference points. More specifically, we hypothesize that (4) when given a static task (“Describe the situation”), the size effect will be larger than the direction effect; and (5) when given a dynamic task (“Describe the change”), the direction effect will exceed the size effect.

## Materials and Methods

### Procedure and Participants

Both experiments were created in Qualtrics. Participants were mostly recruited through Prolific (prolific.com), with the sole requirement that Dutch was their native language. Informed consent was obtained for the experiment following the procedures suggested by the Ethical Committee of Utrecht Institute of Linguistics OTS (approved under #Holle102-01-2018). Experiment 1 (static task) ran in July 2019, Experiment 2 (dynamic task) in April 2020 (excluding participants from Experiment 1). For the static task round, an additional number of 32 participants were found by advertising the experiment in a first-year course of the program Communication and Information Sciences. Prolific participants received a payment based on an hourly rate of Euro 7.00 and an estimated time for the task of 15 min. After cleaning the data, Experiment 1 consisted of 120 participants, and there were 132 participants for Experiment 2.

We cleaned the data by checking the time spent on the task, and by controlling for inconsistent answers. One participant who spent less than 4 min on Experiment 1 was discarded. As answers may be incorrect combinations of frame components and percentages, we checked the number of incorrect answers per participant. Three participants with seven, eight and nine mistakes (out of 16 answers) were discarded from Experiment 1. For this experiment, 20 participants with one incorrect answer, and two participants with two incorrect answers were retained, as these were considered slips; the incorrect items were discarded. Likewise, for Experiment 2 we removed the data of two participants with eight mistakes. We retained 13 participants with only one mistake, and two participants with two mistakes, discarding the incorrect items. Using these criteria, in the total data across the two experiments, 41 out of 4,032 responses were discarded.

In the resulting data set for Experiment 1, 59.2% the participants were men, 40% were women [and 1 (0.8%) participant did not share gender information]. For Experiment 2, these proportions were 63.6, 35.6, and 0.8%, respectively. Generally our participants were young and highly educated. Mean participant age was 27.1 years (SD 9.3) for Experiment 1, and 26.4 (SD 8.2) for Experiment 2. In Experiment 1 81.7% of the participants was highly educated, i.e., having a BA degree or MA degree; in Experiment 2, 85.6% was highly educated. Within experiments, there were no differences between the four lists for participant gender, age or educational level. Neither did we find demographic differences between the participants of Experiment 1 and Experiment 2.

### Experimental Design and Item Lists

Experiment 1 presented the items with a static task (how would you prefer to describe the recent situation) whereas Experiment 2 consisted of a dynamic task (how would you prefer to describe this development). Apart from the task, the two experiments were identical in design, i.e., in items and lists. Both experiments used a 2 (symmetry: asymmetrical/symmetrical) × 2 (direction: the first component is increasing/decreasing) × 2 (size: the first component is the larger/the smaller one) design.

Symmetry varied within participants and between items: an item was either symmetrical or asymmetrical. The direction and size variables varied both within participants and within items; a Latin Square design was used so that each participant saw only one size-direction version of each item. Every participant was presented with 16 experimental items, in which symmetry, direction and size were crossed variables; there were 16 filler items presenting an unrelated task. Participants were randomly allocated to the four experimental lists in Supplementary Table 1, used for both experiments. As can be seen there, size-direction combinations and symmetrical and asymmetrical items were mixed in an irregular pattern.

### Scenarios and Description Tasks

**FIGURE 2:**
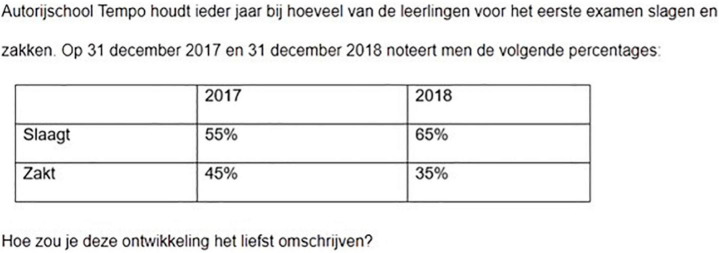
Screenshot of an asymmetrical scenario (taken from experiment 2). Translation: Driving School *Tempo* keep tabs of how many pupils pass and fail their first exam. At the end of 2017 and 2018, the following percentages were given: [..]. How would you prefer to describe this development?

In constructing the experimental items, we wrote little scenarios describing a two-valued distribution that had changed, comparing two points in time. The distributions were presented in 2 × 2 tables. The two time points were consecutive years, presented in the columns. For the final year, we used four distributions, randomly assigned to and evenly spread over the symmetrical and asymmetrical item sets: 65--35%, 70--30%, 75--25%, and 80--20%. That is, the smaller component was never smaller than 20%. The percentages changed over time with either 10% (for the 65--35% and the 80--20% distributions) or 15% (for the 70--30% and the 75--25% distributions)^1^. Figure 1 above and Figure 2 below provide examples using a 65–35% final state and an increment of 10%.

For the asymmetrical items we confined ourselves to the first source of markedness given above, i.e., success-failure asymmetries relating to goal orientations. Such contrasts are most clearly two-valued. Many evaluation (*good-bad*) and perceptual saliency contrasts (*full-empty*) are scalar, and gender is increasingly recognized as a non-binary notion. For the symmetrical items, we constructed pairs which were plausible binary contrasts in the given scenario. For instance, in the Figure 1 scenario above it can be assumed that most animals presented to shelters are either cats or dogs.

Table 1 presents the four distribution conditions for the scenario shown in Figure 2. The conditions are obtained by crossing two factors: size and direction of change. Each subject read one distribution, see Supplementary Table 1. Figure 1 above presents a symmetrical scenario in which the first component (*dogs*) is the decreasing majority component; Figure 2 presents an asymmetrical scenario in which the unmarked component (*passes*) is the increasing majority component.

**TABLE 1:** Four versions of the distribution used in Figure 2.

	Largest – increasing		Largest – decreasing		Smallest – increasing		Smallest – decreasing	
	2017	2018	2017	2018	2017	2018	2017	2018
*Passes*	55%	65%	75%	65%	25%	35%	45%	35%
*Fails*	45%	35%	25%	35%	75%	65%	55%	65%

**FIGURE 3:**
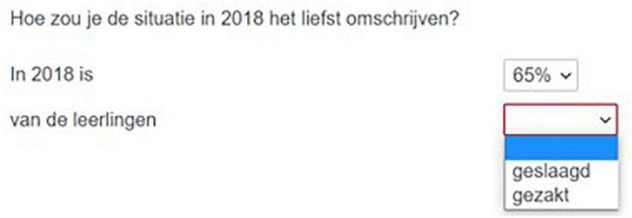
Screen shot of the answering format in the static task (experiment 1). Translation: How would you prefer to describe the situation in 2018? In 2018 (35%) 65% of the pupils … passed/failed. Note: this translation is a gloss of the Dutch word order.

**FIGURE 4:**
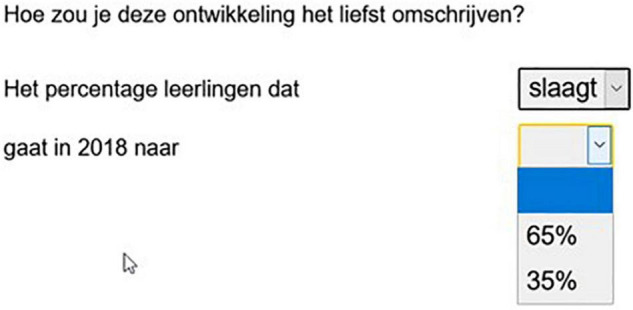
Screen shot of the answering format in the dynamic task (experiment 2). Translation: how would you prefer to describe this development? The percentage of pupils passing (failing) develops in 2018 into. 65/35%. Note: this translation is a gloss of the Dutch word order.

We used two description tasks: participants were asked to describe what the most recent situation looks like (static task, Experiment 1) or what has changed (dynamic task, Experiment 2). Figures 3, 4, respectively, provide the static and dynamic task for the Figure 2 item.

After reading *“How would you prefer to describe this development?”* the participants completed the sentence *“The percentage of pupils that…”* by clicking on the component of their choice (*passes* or *fails*), and then choosing the corresponding percentage. Compare the screen shot in Figure 4, which shows that even though the *pass-*frame (Dutch *slaagt*) has already been chosen, both percentage options are still shown. This second choice requires the participant to actually process the scenario information before responding.

In the first choice between components, we did not vary the order of answering options. The options for asymmetrical pairs were offered in the most natural order, i.e., the unmarked component was mentioned first. Starting with the failure frame would have surprised some participants. Hence the order of the symmetrical pairs was also fixed, by following the alphabetic order (in Dutch) of the pair members. For all items, the probability of choosing the component presented first served as the dependent variable. Given a certain primacy effect, these percentages may exceed 50%, also for symmetrical items. However, this does not invalidate our data. First, in an earlier framing interpretation study which counterbalanced orders of answering options (Holleman and Pander Maat, 2021), we found no interaction between order and the variables of interest. Second, there is no conceptual argument for assuming an interaction between symmetry and answering order. More specifically, it is unclear why the unmarked option in asymmetrical items would show a higher primacy benefit than the first component in symmetric pairs – in fact, the opposite seems more likely: for symmetrical items, primacy is the only advantage of the first option, an advantage undiluted by an additional unmarkedness preference. In sum, the cost of counterbalancing answering options would exceed the benefit.

### Hypotheses and Statistical Analysis

Below, we will report an analysis of each experiment separately. Three hypotheses apply to both experiments:

1. There is a main effect of Size in that largest components will be framed more often.
2. There is a main effect of Direction in that increasing components will be framed more often.
3. There is a main effect for Asymmetry, in that asymmetrical items will show more choices for the first component, as this is the unmarked one.

Hypotheses 1–3 were tested by means of a generalized mixed model analysis in SPSS (binomial distribution, Logit link), with choice for the first frame component as the dichotomous dependent variable. Item and participants were entered as crossed random factors, with random intercept and no random slopes. We tested a complete factorial model containing all main effects and interactions. In case of interactions, the hypotheses are checked using pairwise contrasts.

Our critical hypotheses distinguish between the two experiments.

4. In Experiment 1 (static task), the Size effect will be larger than the Direction effect.
5. In Experiment 2 (dynamic task), the Direction effect will be larger than the Size effect.

Hypotheses 4–5 will be tested by checking overlap between the confidence intervals for the two effects (note that comparing two 95% confidence intervals is a very conservative significance test, see Goldstein and Healy, 1995). In case of interactions, the significance of single pairwise contrasts is tested by reviewing whether 95% confidence intervals overlap with 0.

## Results

### Experiment 1 (Static Task)

We found a primacy effect for component order: for symmetrical items, 56.9% of the choices went to the firstly mentioned component. Supplementary Table 2 shows the choice percentages of the firstly mentioned component for individual items in Experiment 1. Symmetrical items 1 (dogs/cats) and 12 (strawberries/oranges) show a stronger bias toward the firstly mentioned component. In asymmetrical items, the first component benefits both from a primacy effect as well as the preference for unmarked pair members. Hence, the first component percentages are generally higher than those in the symmetrical items (63.9%). Only asymmetrical item 28, about finishing or dropping out of a hazing ritual deviates strongly with a score of 48.3%. It could be that scenario-specific expectations are at stake here. As the goal of a hazing ritual (from the perspective of the hazers) is to give participants a hard time, it’s possible that the framing choice of “finishing” is less unmarked than the success component in other scenario’s.

All this shows that item-specific factors affect component choice. However, this does not threaten our study: the multilevel analysis takes into account variances between individual items, as item is one of the random factors in the analysis. Significant fixed effects in such an analysis indicate that the effect generalizes over items. This is especially meaningful when dealing with items showing individual peculiarities. Hence we will further focus on this analysis. Table 2 shows main effects for Size, Direction and Symmetry, qualified by a Symmetry × Size interaction and a three-way interaction.

**TABLE 2:** Fixed effects analysis for experiment 1.

Fixed effect term	Coefficient	*SE*	*t*	*p*	*Exp*
Intercept	–1.61	0.23	–6.93	<0.001	0.20
**Size (largest-smallest)**	3.56	0.26	13.66	<0.001	35.04
**Direction (increasing-decreasing)**	0.63	0.23	2.77	<0.01	1.88
**Symmetry (asymmetric-symmetric)**	1.02	0.30	3.41	<0.01	2.78
Symmetry × Direction	–0.05	0.30	–0.16	n.s.	0.95
**Symmetry × Size**	–1.00	0.35	–2.83	<0.01	0.37
Size × Direction	0.39	0.41	0.95	n.s.	1.48
**Symmetry × Size × Direction**	–1.31	0.53	–2.46	<0.05	0.27

**Random effect term**	***Estimate***	***SE***	***Z***	***p***	

Participants^+^	0.50	0.13	3.92	<0.001	
Item^+^	0.16	0.08	1.94	n.s.	

*Bolded fixed effect terms show significant effects.*

*SE, standard error; Exp, exponentiated coefficient (odds ratio).*

*^+^The structure of the variance-covariance matrix is variance components.*

**FIGURE 5:**
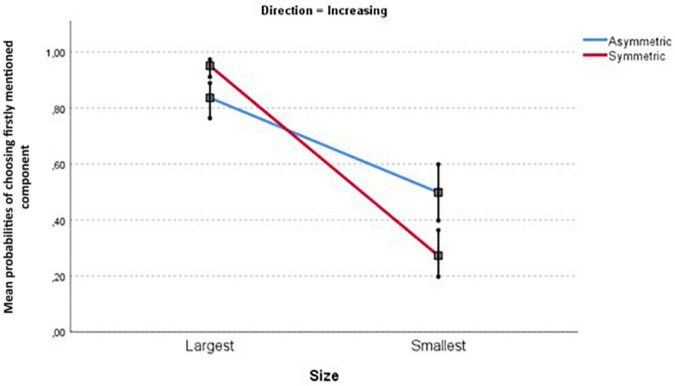
Mean probabilities for the Size × Symmetry contrast for increasing proportions (see rows 1–4 in Table 3). Note: I indicate 95% confidence intervals.

**FIGURE 6:**
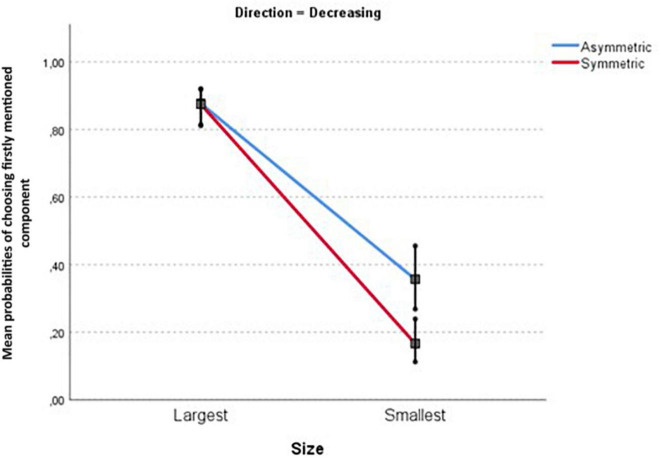
Mean probabilities for the Size × Symmetry contrast for decreasing proportions (see rows 5–8 in Table 3). I indicate 95% confidence intervals. Please note that the confidence intervals for the two conditions of decreasing-largest-symmetric (Lower CI = 0.812; Upper CI = 0.919) and decreasing-largest-asymmetric (Lower CI = 0.814; Upper CI = 0.920) can not be visually distinguished from each other.

These interaction effects necessitate further pairwise comparisons. Table 3 and Figures 5, 6 unpack this interaction.

**TABLE 3:** Probabilities involved in the three-way interaction in experiment 1.

	Direction	Symmetry	Size	Mean prob.	95% CI pairwise contrast *size*	95% CI pairwise contrast *direction*	95% CI pairwise contrast *symmetry*
1.	Increasing	Asymmetric	Largest	0.837	Row 1–2: 0.248–0.428	Row 1–5: n.s.	Row 1–3: (–0.047)–(–0.182)
2.	Increasing	Asymmetric	Smallest	0.499		Row 2–6: 0.048–0.237	Row 2–4: 0.101–0.351
3.	Increasing	Symmetric	Largest	0.951	Row 3–4: 0.600–0.757	Row 3–7: 0.24–0.129	
4.	Increasing	Symmetric	Smallest	0.272		Row 4–8: 0.030–0.183	
5.	Decreasing	Asymmetric	Largest	0.877	Row 5–6: 0.435–0.605		Row 5–7: n.s.
6.	Decreasing	Asymmetric	Smallest	0.356			Row 6–8: 0.081–0.300
7.	Decreasing	Symmetric	Largest	0.875	Row 7–8: 0.645–0.772		
8.	Decreasing	Symmetric	Smallest	0.166			

The following main results can be read from Table 3:

– Large components are consistently preferred over small ones (column “contrast size”). The minimal large-small difference is about 84 vs. 50% (increasing components in asymmetric items), the maximal difference is 88 vs. 17% (decreasing components in symmetric items).
– In three out of four comparisons, increasing components are preferred over decreasing ones; the differences range from 95 vs. 88% (largest components in symmetric items) to 50 vs. 36% (small components in asymmetric items). But for large components in asymmetric contrasts, there is no difference (rows 1–5).
– As mentioned earlier, all probabilities refer to the component mentioned first. In asymmetrical contrasts, this concerns the unmarked pair member (e.g., *winning* instead of *losing*). Hence we expected higher probabilities for asymmetric items than for symmetric ones. This difference was only found for small components, with both differences around 20%. For large components there is either no symmetry difference, or a tendency to prefer the marked pair member (e.g., *losing*) instead of the unmarked one (e.g., *winning*).

These patterns already suggests that the Size effect is the most consistent one in this task: participants tend to frame the description of the situation in terms of the largest component. We tested Hypothesis 4 (the Size effect is stronger than the direction of change effect) by examining the confidence intervals for the contrasts (i.e., the pairwise differences) involved. As expected, the Size contrast intervals (see Table 3) are consistently larger than those for the three significant Direction contrasts.

The interplay between Size, Direction and Symmetry is visualized in Figures 5, 6. We find an overall preference to frame in terms of the largest component, both for increasing and decreasing components. Furthermore, we see smaller Size effects for asymmetric contrasts in both figures. Separate analyses show that this Size × Symmetry interaction remains significant for both increasing components (coefficient = −2.235, *SE* = 0.4022, *t* = −5.557, *p* < 0.001) and decreasing components (coefficient = −0.980, *SE* = 0.3483, *t* = −2.812, *p* < 0.01).

Summarizing, a three-way interaction in Experiment 1 required us to test the hypotheses by means of four pair-wise comparisons. These consistently support Hypothesis 1: with the static task in this experiment, there is an across-the-board Size effect. Only three comparisons show Direction effects, and only two show Symmetry effects, against Hypotheses 2 and 3. Crucially, the results support Hypothesis 4, in that Size effects are consistently larger than Direction effects (when present).

### Experiment 2 (Dynamic Task)

Again, we found a primacy effect for component order: for symmetrical items, 58.1% of the choices went to the firstly mentioned component; for asymmetrical items, this was 71.7%. Supplementary Table 2 provides the item scores, again showing some peculiarities. As explained earlier, we use a multilevel analysis to take into account the variance between individual items.

The analyses were focused on the hypotheses discussed in Section “Hypotheses and statistical analysis.” In Experiment 2, we only found main effects. The significant positive coefficients in Table 4 reveal that our participants consistently prefer the largest component (size effect), the increasing component (direction effect) and the unmarked component (Symmetry effect, i.e., the first pair member was more often chosen in asymmetric contrasts than in symmetric contrasts). These findings support Hypotheses 1, 2, and 3, respectively.

**TABLE 4:** Fixed effects analysis for experiment 2.

Fixed effect term	Coefficient	*SE*	*t*	*p*	*Exp*
Intercept	–0.74	0.17	–4.40	<0.001	0.48
**Size (largest-smallest)**	0.61	0.19	3.25	<0.01	1.83
**Direction (increasing-decreasing)**	1.56	0.19	8.08	<0.001	4.77
**Symmetry (asymmetric-symmetric)**	0.99	0.22	4.51	<0.001	2.70
Symmetry × Direction	–0.40	0.28	–1.44	n.s.	0.67
Symmetry × Size	–0.30	0.26	–1.15	n.s.	0.74
Size × Direction	0.35	0.29	1.19	n.s.	1.41
Symmetry × Size × Direction	0.13	0.43	0.31	n.s.	1.14

***Random effect term***	***Estimate***	***SE***	***Z***	***p***	

Participants^+^	0.44	0.10	4.20	<0.001	
Item^+^	0.06	0.04	1.51	n.s.	

*Bolded fixed effect terms are significant.*

*SE, standard error; Exp, exponentiated coefficient (odds ratio).*

*^+^The structure of variance-covariance matrix is variance components.*

Hypothesis 5 was supported by the confidence intervals surrounding the contrast estimates for the Size and Direction effects; see Tables 5, 6: the Direction effect (82 vs. 50%; CI = 29–37%) is clearly larger than the Size effect (75 vs. 61%; CI = 10–19%). As the communicative task in Experiment 2 focuses on the development between the two time points, direction of change is more important than size.

**TABLE 5:** The size contrast in experiment 2.

Size	Mean probability	Contrast value	95% CI contrast
Largest	0.749	0.142	0.098–0.187
Smallest	0.607		

**TABLE 6:** The direction contrast in experiment 2.

Direction	Mean probability	Contrast value	95% CI contrast
Increasing	0.825	0.330	0.286–0.373
Decreasing	0.495		

## Conclusion and Discussion

In two experiments, we studied the influence of component size and component change on attribute framing choices, and the role of the symmetry of the framing contrast. While we expected preferences for larger and increasing components, our crucial argument was that the relative strength of these preferences would be determined by the static or dynamic focus of the framing production task. More specifically, we expected the Size effect to be more important in the static description task of Experiment 1 and the Direction-of-change effect to be more important in the dynamic task of Experiment 2.

Experiment 1 supported the expectation that in the static task, large components are consistently preferred. Direction-of-change effects appear in three out of four pair-wise comparisons, but not for large components in asymmetric contrasts. Crucially, the Size effects were consistently larger than the Change effects, where these appear. In Experiment 1, Symmetry effects are restricted to small components, i.e., components whose significance is not already boosted by the Size effect dominating the choices in the static task. Furthermore, the Size effect is somewhat reduced for asymmetrical contrasts, i.e., the preference for the unmarked contrast member can diminish (but not eliminate) the preference to frame in terms of the largest component.

In Experiment 2, our dynamic description task showed all three hypothesized main effects: we find a preference to frame in terms of the largest pair member, and a preference to frame in terms of the increasing pair member, as well as a preference to frame in terms of the unmarked pair member. Crucially, the dynamic task shows the expected reversal of effects compared to Experiment 1 with the static task: while the Size effect prevails in the static task, in the dynamic task the Change effect is much stronger.

In sum, our study shows Size effects regardless the task. Direction of change effects are somewhat less robust, in that they fail to emerge in one of the comparisons in Experiment 1. Taken together, frame producers do tend to choose large and increasing components. The essential finding of this study is that the relative strength of both effects depends on the frame production task. When asked about the situation at a certain time point, language users primarily use the parity reference point and focus on the largest component; when asked about a development, the previous state reference point reigns and language users focus on growth. All this holds across a variety of scenarios. It also applies to both symmetric and asymmetric contrasts, although the Size effect in static tasks is reduced (not eliminated) in asymmetric scenarios. Finally, we find that Symmetry effects may be overridden by Size effects in static tasks.

More generally, we have shown that framing choice is not solely determined by the distributions given for a certain scenario, but by these distributions *in combination with* particular communicative contexts; these contexts were operationally defined by asking different questions about the distributions. Such a question makes certain utterances more relevant than others, and we have proposed that reference points correspond to types of relevance. In other words, static descriptions’ primary relevance lies in communicating that the parity reference point is exceeded, while the point of dynamic descriptions is that some component goes beyond the previous state reference point. This link between reference points and relevance shows how in attribute framing, cognitive representations are geared toward situated communicative action.

Of course, the two types of relevance now demonstrated by no means exhaust the communicative affordances provided by framing choices (or linguistic construals more generally). In certain contexts, framing choices convey Argumentative Orientations regarding evaluations or recommendations (Holleman and Pander Maat, 2009). Smart experimental designs are needed to further disentangle how input information and communicative considerations co-determine frame choices. Our study makes a start by providing frame producers with different descriptive foci; in the future, more radical communicative goal variations (e.g., describing a situation vs. persuading the reader) may be studied as well (Van Buiten and Keren, 2009).

Communicative aspects of framing also need to be explored further in frame interpretation studies, in addition to frame construction studies such as this one. Given that previous state inferences have now been documented abundantly (Sher and McKenzie, 2006; Honda et al., 2018; Holleman and Pander Maat, 2021), we would recommend to focus on hearer inferences related to frame component sizes. Given the consistent size effects across tasks, descriptions in terms of small components can be expected to generate specific inferences. This is comparable to what has been found for descriptions using marked (i.e., dispreferred) pair members: Holleman and Pander Maat (2009) have shown that a description in terms of *losses* suggests more negativity than a *win-*frame conveys positivity. Similarly, we expect that a situation described in terms of the smallest component will lead to stronger inferences either in terms of previous states (i.e., the suggestion that the component has increased will be stronger for small than for large components) or in terms of projected conclusions [as it is suggested that this (low) number is especially relevant in line of the conclusion to be drawn about this situation].

We also suggest further research into other types of reference points. Besides the two kinds of reference points at issue here, at least one other kind of reference point has been demonstrated, based on scenario-specific frequency expectations. For instance, Experiment 4 in Sher and McKenzie (2006) used loaded dice to manipulate expectations, which were shown to affect frame choices. Similarly, Honda and Matsuka (2014) have shown how information on the rarity of events may influence frame choices. Generally, components for which the sizes exceeds the expectation, will be framed more often than the complementary ones, regardless the source of the expectation.

Another source of reference points might be normative expectations. For instance, when talking about the number of men and women in particular professions, one might be focused on underrepresentation of women (for instance, the number of women being less than 50%). With regard to such normative reference points, the normal preference to frame quantities exceeding the reference points might not apply; instead, one might prefer to frame components that *fail* to reach the expected levels. Alternatively, such cases could be accounted for in terms of markedness contrasts stemming from goal orientations. That is, in many gender equality scenarios, *women* are the desired and hence unmarked component, like the number of games *won* is in competition situations.

Finally we should note a limitation of this work, though one inevitable in the current state of the field. In order to demonstrate the reference points, we have severely restricted the utterances of our participants: our description instructions were highly specific, our response templates tightly constrained. Future studies could think of more open formats, or study natural language use in corpora, in order to broaden our understanding of the ways reference points steer both production and interpretation of framed messages. Such studies, along with the present one, are needed to further uncover the linguistic options available in framed utterances, and the communicative logic behind them.

## Data Availability Statement

The datasets presented in this study can be found in online repositories. The names of the repository/repositories and accession number(s) can be found below: https://doi.org/10.17026/dans-zm6-u9d9.

## Ethics Statement

The studies involving human participants were reviewed and approved by https://fetc-gw.wp.hum.uu.nl. The patients/participants provided their written informed consent to participate in this study.

## Author Contributions

HPM and BH contributed to the conception and the design of the study, wrote the manuscript. HPM, BH, and BS constructed the materials. BS organized the data collection. HPM and BS conducted the statistical analyses. All authors contributed to manuscript revision, read, and approved the submitted version.

## Conflict of Interest

The authors declare that the research was conducted in the absence of any commercial or financial relationships that could be construed as a potential conflict of interest.

## Publisher’s Note

All claims expressed in this article are solely those of the authors and do not necessarily represent those of their affiliated organizations, or those of the publisher, the editors and the reviewers. Any product that may be evaluated in this article, or claim that may be made by its manufacturer, is not guaranteed or endorsed by the publisher.

## Footnotes

1 Koo and Fishbach (2012) have shown that when monitoring task progress, saying “20% has been done” is more motivating than saying “80% still needs to be done.” They explain this in terms of the relative size of the next step in the task. For instance, if that the next step adds 10%, this step appears as larger in the “20% done” context than in the “80% to be done” context. Our task is entirely different, but it might be that increment size is a factor in our study as well. Hence we checked whether the size of our changes (either 10 or 15%) affected framing choices. However, we found no reliable increment size effects. Given the results to be reported later on, this indicates that in our task, direction of change is essential, rather than the size of the change.
