# Supplementary material for: The Framing Preference for Large and Increasing Components in Static and Dynamic Descriptions
Source: Front Psychol. 2021 Nov 18;12:720427. doi: 10.3389/fpsyg.2021.720427 (PMC8636774; doi:10.3389/fpsyg.2021.720427)
Supplement: Supplementary file 1 [file Data_Sheet_1.docx]

Supplementary Material

**Supplement 1. Experimental lists (for both tasks)**

*Item class abbreviations*

Asym – G – I: asymmetrical / largest / increasing

Asym – G – D: asymmetrical / largest / decreasing

Asym – Sm – I: asymmetrical / smallest / increasing

Asym – Sm – D: asymmetrical / smallest / decreasing

Sym – G – I: symmetrical / largest / increasing

Sym – G – D: symmetrical / largest / decreasing

Sym – Sm – I: symmetrical / smallest / increasing

Sym – Sm – D: symmetrical / smallest / decreasing

| **Item**  **number** | **Experimental / filler item** | **Scenario topic** | **List 1** | **List 2** | **List 3** | **List 4** |
| --- | --- | --- | --- | --- | --- | --- |
| 1 | Experimental | Dogs / cats brought to an animal shelter | Sym-G-I | Sym-G-D | Sym-Sm-I | Sym-Sm-D |
| 2 | Filler |  |  |  |  |  |
| 3 | Filler |  |  |  |  |  |
| 4 | Experimental | Mars / Snickers as the snack of choice during the break in a hockey match | Sym-Sm-D | Sym-G-I | Sym-G-D | Sym-Sm-I |
| 5 | Filler |  |  |  |  |  |
| 6 | Experimental | Passing / failing pupils of driving school | Asym-G-I | Asym-G-D | Asym-Sm-I | Asym-Sm-D |
| 7 | Experimental | Cows / sheep as a proportion of cattle on a farm | Sym-G-D | Sym-Sm-I | Sym-Sm-D | Sym-G-I |
| 8 | Filler |  |  |  |  |  |
| 9 | Experimental | Won / lost judo matches | Asym-Sm-D | Asym-G-I | Asym-G-D | Asym-Sm-I |
| 10 | Filler |  |  |  |  |  |
| 11 | Filler |  |  |  |  |  |
| 12 | Experimental | Strawberries / oranges as fruit drink ingredients | Sym-G-I | Sym-G-D | Sym-Sm-I | Sym-Sm-D |
| 13 | Filler |  |  |  |  |  |
| 14 | Experimental | Body lotion / douche gel as the chosen welcome present in a tanning salon | Sym-Sm-I | Sym-Sm-D | Sym-G-I | Sym-G-D |
| 15 | Experimental | Won / lost matches of a volleyball team | Asym-G-D | Asym-Sm-I | Asym-Sm-D | Asym-G-I |
| 16 | Filler |  |  |  |  |  |
| 17 | Experimental | Scored / missed penalty kicks in children’s soccer tournament | Asym-G-I | Asym-G-D | Asym-Sm-I | Asym-Sm-D |
| 18 | Filler |  |  |  |  |  |
| 19 | Filler |  |  |  |  |  |
| 20 | Experimental | Number of participants winning a price / no price in the lottery | Asym-Sm-I | Asym-Sm-D | Asym-G-I | Asym-G-D |
| 21 | Filler |  |  |  |  |  |
| 22 | Experimental | Chicken soup / tomato soup a menu choices of elderly care clients | Sym-Sm-I | Sym-Sm-D | Sym-G-I | Sym-G-D |
| 23 | Experimental | Men / women among first-year Law students | Sym-G-D | Sym-Sm-I | Sym-Sm-D | Sym-G-I |
| 24 | Filler |  |  |  |  |  |
| 25 | Experimental | Passing / failing integration tests by immigrants | Asym-Sm-D | Asym-G-I | Asym-G-D | Asym-Sm-I |
| 26 | Filler |  |  |  |  |  |
| 27 | Filler |  |  |  |  |  |
| 28 | Experimental | Finishing / dropping out from the hazing ritual in a student fraternity | Asym-Sm-I | Asym-Sm-D | Asym-G-I | Asym-G-D |
| 29 | Filler |  |  |  |  |  |
| 30 | Experimental | Choosing hockey / softball on a school sports day | Sym-Sm-D | Sym-G-I | Sym-G-D | Sym-Sm-I |
| 31 | Experimental | Arrows hitting / missing the target in a bow and arrow competition | Asym-G-D | Asym-Sm-I | Asym-Sm-D | Asym-G-I |
| 32 | Filler |  |  |  |  |  |

**Supplement 2. Choice percentages of firstly mentioned component for each item in both experiments, sorted by symmetry**

| **Item**  **number** | **Symmetry** | **Scenario topic** | **Choice percentages for the firstly mentioned component in the static task (experiment 1)** | **Choice percentages for the firstly mentioned component in the dynamic task (experiment 2)** |
| --- | --- | --- | --- | --- |
| 1 | symmetrical | Dogs / cats brought to an animal shelter | 65.3% | 66.7% |
| 4 | symmetrical | Mars / Snickers as the snack of choice during the break in a hockey match | 54.6% | 52.7% |
| 7 | symmetrical | Cows / sheep as a proportion of cattle on a farm | 57.5% | 55.7% |
| 12 | symmetrical | Strawberries / oranges as fruit drink ingredients | 62.5% | 75.5% |
| 14 | symmetrical | Body lotion / douche gel as the chosen welcome present in a tanning salon | 57.1% | 60.0% |
| 22 | symmetrical | Chicken soup / tomato soup a menu choices of elderly care clients | 47.9% | 55.0% |
| 23 | symmetrical | Men / women among first-year Law students | 53.4% | 50.8% |
| 30 | symmetrical | Choosing hockey / softball on a school sports day | 56.7% | 61.1% |
| Mean |  |  | 56.9% | 58.1% |
| 6 | asymmetrical | Passing / failing pupils of driving school | 64.6% | 69.5% |
| 9 | asymmetrical | Won / lost judo matches | 70.9% | 73.5% |
| 15 | asymmetrical | Won / lost matches of a volleyball team | 66.9% | 75.8% |
| 17 | asymmetrical | Scored / missed penalty kicks in children’s soccer tournament | 61.0% | 79.4% |
| 20 | asymmetrical | Number of participants winning a price / no price in the lottery | 78.2% | 76.2% |
| 25 | asymmetrical | Passing / failing integration tests by immigrants | 56.3% | 68.2% |
| 28 | asymmetrical | Finishing / dropping out from the hazing ritual in a student fraternity | 48.3% | 59.8% |
| 31 | asymmetrical | Arrows hitting / missing the target in a bow and arrow competition | 65.5% | 71.2% |
| Mean |  |  | 63.9% | 71.7% |
